# Role of the prostaglandin E2/E-prostanoid 2 receptor signalling pathway in TGFβ-induced mice mesangial cell damage

**DOI:** 10.1042/BSR20140130

**Published:** 2014-12-12

**Authors:** Na-Na Li, Yu-Yin Xu, Xiao-Lan Chen, Ya-Ping Fan, Jian-Hua Wu

**Affiliations:** *Department of Nephrology, Affiliated Hospital of Nantong University, Nantong, Jiangsu, China

**Keywords:** adenovirus, EP2, ERK1/2, PGE_2_, siRNA, TGFβ1, BP, blood pressure, CCK, cholecystokinin, CKD, chronic kidney disease, Col I, collagen type I, COX2, cyclooxygenase-2, CRE, CREB, cAMP responsive element binding protein, CTGF, connective tissue growth factor, DMEM, Dulbecco’s modified Eagle’s medium, ECM, extracellular matrix, EP2, E-prostanoid 2, ERK, extracellular-signal-regulated kinase, FBS, fetal bovine serum, FN, fibronectin, GAPDH, glyceraldehyde-3-phosphate dehydrogenase, JNK, c-Jun N-terminal kinase, MAPK, mitogen-activated protein kinase, MC, mesangial cell, MOI, multiplicity of infection, mPGES-1, membrane associated prostaglandin E1, PGE_2_, prostaglandin E_2_, PGES, prostaglandin E_2_ synthase, PKA, protein kinase A, RT–PCR, reverse transcription–PCR, siRNA, small interfering RNA, TGFβ1, transforming growth factor-β1

## Abstract

The prostaglandin E2 receptor, EP2 (E-prostanoid 2), plays an important role in mice glomerular MCs (mesangial cells) damage induced by TGFβ1 (transforming growth factor-β1); however, the molecular mechanisms for this remain unknown. The present study examined the role of the EP2 signalling pathway in TGFβ1-induced MCs proliferation, ECM (extracellular matrix) accumulation and expression of PGES (prostaglandin E_2_ synthase). We generated primary mice MCs. Results showed MCs proliferation promoted by TGFβ1 were increased; however, the production of cAMP and PGE_2_ (prostaglandin E_2_) was decreased. EP2 deficiency in these MCs augmented FN (fibronectin), Col I (collagen type I), COX2 (cyclooxygenase-2), mPGES-1 (membrane-associated prostaglandin E1), CTGF (connective tissue growth factor) and CyclinD1 expression stimulated by TGFβ1. Silencing of EP2 also strengthened TGFβ1-induced p38MAPK (mitogen-activated protein kinase), ERK1/2 (extracellular-signal-regulated kinase 1/2) and CREB1 (cAMP responsive element-binding protein 1) phosphorylation. In contrast, Adenovirus-mediated EP2 overexpression reversed the effects of EP2-siRNA (small interfering RNA). Collectively, the investigation indicates that EP2 may block p38MAPK, ERK1/2 and CREB1 phosphorylation via activation of cAMP production and stimulation of PGE_2_ through EP2 receptors which prevent TGFβ1-induced MCs damage. Our findings also suggest that pharmacological targeting of EP2 receptors may provide new inroads to antagonize the damage induced by TGFβ1.

## INTRODUCTION

The progression of CKD (chronic kidney disease) is characterized by glomerular hypertrophy, MC (mesangial cell) proliferation, ECM (extracellular matrix) accumulation, glomerulosclerosis and ultimately ESKD (end-stage kidney disease) [1]. Central to the pathophysiology of CKD are the MCs. ECM is produced by damaged MCs and contains collagens type I, IV and V, laminin A, B1 and B2, FN and entactin, and nidogen. ECM is a major factor of mesangial expansion as seen in light-chain-related glomerular disease associated with increased synthesis of tenascin by the MCs [2].

TGFβ has been recognized as a central player in many pathological events related to CKD progression at the glomerular, tubulointerstitial and vascular levels. Increased TGFβ expression in the obstructed kidney stimulates genes involved in ECM protein accumulation, including type 1 collagen and FN [3]. Additionally, TGFβ stabilizes the ECM proteins by stimulating the expression of PAI-1 (plasminogen activator inhibitor 1). Thus, the inhibition of TGFβ signalling has been included in several therapeutic approaches for preventing renal fibrosis.

Prostaglandin mainly PGE_2_ (prostaglandin E_2_) plays an important role in renal haemodynamics, renin release and salt and water homoeostasis. The physiological effects of PGE_2_ are mediated through prostaglandin E receptors, called EP receptors. Four EP receptors (EP1 to EP4) are currently known. They are all G-protein-coupled seven-transmembrane receptors, with a strong heterogeneity with respect to the underlying signal transduction pathways. Stimulation of the EP1 receptor results in activation of PI (phosphatidylinositol) hydrolysis and elevation of intracellular Ca^2+^ concentration. [4] EP2 (E-prostanoid 2) and EP4 receptors coupled to Gs, and activation of these receptors results in stimulation of adenyl cyclase and increases intracellular cAMP [5]. The major signalling pathway described for the EP3 receptor is mediated by Gαi and leads to a reduction in intracellular cAMP levels [6]. Thus PGE_2_ may cause different intracellular responses depending on the specific distribution of the different EP receptors on a particular cell type.

MAPK (mitogen-activated protein kinase) signal transduction pathways are evolutionarily conserved among eukaryotes. Transduction of the extracellular stimuli to the nucleus for gene activation is primarily conducted through the type-dependent stimulation of kinases in the three MAPK subfamilies as follows: ERK (extracellular-signal-regulated kinase), JNK (c-Jun N-terminal kinase) and p38. They have been implicated in a number of biological processes, including cell growth, differentiation, apoptosis, inflammation and responses to environmental stresses [7,8].

The present research on PGE_2_ receptors, EP1–EP4 is mainly focused on their roles in the regulation of BP (blood pressure) through sodium–water homoeostasis. The studies suggest that the COX2/PGES1/PGE_2_/EP2 pathway in the renal medulla plays an important role in modulating sodium excretion and maintaining body sodium balance and systemic BP [9,10]. Among the four EPs, EP3 and EP4 receptors are the most widely distributed with their mRNAs being expressed in almost all mouse tissues examined. In contrast, the distribution of EP1 mRNA is restricted to several organs, such as the kidney, lungs, and stomach, and EP2 is the least abundant of the EP receptors [11]. Although only low levels of EP2 mRNA was detected in the kidney, when fed a diet high in salt, the EP2^−/−^ mice developed profound systolic hypertension, whereas wild-type mice showed no change in systolic BP [12].

At present, the role of the EP2 in mediating renal fibrosis in CKD remains unknown. In the study, EP2–siRNA (small interfering RNA) and AD-EP2 were used to decrease or elevate EP2 expression in primary wild-type MCs. The aim of the present study is to determine the possible mechanism by which EP2 receptors affect the activities induced by TGFβ1 in MCs.

## MATERIALS AND METHODS

### Animals

C57/BL6 mice aged between 8 and 12 weeks were provided by Animal Experimentation Committee of Beijing University. All mice were fed with animal food and kept in an air-conditioned facility at Association for Assessment and Accreditation of Lab Animal Care accredited. The animals were allowed free access to water and food. Welfare-related assessment and interventions were carried out during the experiment. The mice were killed under ether anaesthesia.

### Cell culture

Primary mice MCs were cultured. In brief, kidneys from 8-to 12-week-old male C57BL/6 mice were obtained. Glomeruli were purified from the renal cortex tissue and the resultant glomeruli suspension was digested for 40 min at 37 °C with type I collagenase. Glomeruli were then collected through 70 and 40 μm stainless steel sieves. The digested samples were then centrifuged at 1000 rev./min for 5 min, and the precipitate was re suspended in growth medium [DMEM (Dulbecco's modified Eagle's medium) supplemented with 20% (v/v) FBS, fetal bovine serum (Gibco, Invitrogen)]. The cells were cultured at 37 °C in a humidified incubator containing 5% (v/v) CO_2_. Only those cells at passages 8–10 were used. Then the primary mice MCs were treated differently.

### siRNA transfection

21-Nucleotide RNAs were chemically synthesized by GenePharma Co., Ltd. The siRNA sequence targeting mouse EP2 were as follows: EP2-siRNA-1(5′-CUGGCCAUUAUGACCAUCATT-3′), EP2-siRNA-2(5′-CCUGCAACAUCAGCGUUAUTT-3′) and EP2-siRNA-3(5′-CUGGCUUCAUAUUCAAGAATT-3′). NC siRNA is a random siRNA provided by GenePharma Co., Ltd. The day before transfection, cells were trypsinized and 3×10^5^ cells were seeded in six-well plates. Transient transfection of siRNA was carried out using Lipofectamine™ (Invitrogen). DMEM (100 μl) (Invitrogen) and 10 μl Lipofectamine™ per well were preincubated for 5 min at room temperature. During the incubation period, 100 μl DMEM was mixed with 10 μg siRNA. The two mixtures were combined and incubated for 20 min at room temperature for the complex formation. After incubation, the entire mixture was added to the cells in a final concentration of 300 nM siRNAs. 12 h later, the culture media 20% FBS and 10 ng/ml TGFβ1 were added and the cells were assayed 36 h after transfection. The MCs were divided in five groups: Control; TGFβ1; NC-siRNA+TGFβ1; EP2-siRNA+TGFβ1 and EP2-siRNA.

### Adenoviral transfection

For overexpression studies, replication-deficient recombinant adenovirus (Ad5-CMV) encoding mouse EP2 (Ad-EP2) or control vector [Ad-GFP (green fluorescent protein)] was obtained from Shanghai GenePharma Co., Ltd. On day 3 of differentiation, MCs were transduced with 0.5, 2.5, 5 and 12.5 MOI (multiplicity of infection) of respective viruses for 24 h in serum-free media. The complete media was replaced for an additional 4 days post-infection prior to experimentation.

### Cell proliferation assay

Cell proliferation was quantified by CCK-8 (cholecystokinin-8) assay kit (Dojindo). MCs (3×10^3^) were seeded in 96-well plates in DMEM with 10% FBS, As the cells reached 70–80% confluence, they were transfected with the siRNAs and adenovirus, then exposed to 10 ng/ml TGFβ1 for 24 h in 20% FBS. We exchanged fresh DMEM gently and 10 μl of CCK-8 solution was added to each well, and the plates were incubated for 2 h at 37 °C. We measured the absorbance at 450 nm using aMicroplate Reader 550 (Bio-Rad).

### ELISA assay

MCs (3×10^3^) were seeded in 96-well plates in DMEM with 10% FBS, As the cells reached 70–80% confluence, they were transfected with the siRNAs and adenovirus, then exposed to 10 ng/ml TGFβ1 for 24 h in 20% FBS. The control group only received 0.1% (w/v) BSA. The cell supernatant was then collected. The amount of cAMP and PGE_2_ in the cell supernatant samples was quantified using an Alpha screen cAMP Assay Kit and PGE_2_ Assay Kit (PerkinElmer), respectively, according to the manufacturer's instructions.

### Real time RT–PCR (reverse transcription–PCR)

Total RNA was isolated with PicoPure RNA isolation Kit (Arcturus Bioscience) according to the manufacturer's instructions. The PCR primers were designed by Shanghai Invitrogen Co., Ltd (Table 1). Total RNA was reverse transcribed to cDNA using TaqMan Reverse Transcription Reagents kit (Applied Biosystems) according to the manufacturer's protocol. Real-time PCR was performed at the following conditions: 50 °C 2 min→95 °C 10 min→(95 °C 15 s→60 °C 60 s)×40. Each sample was analysed in triplicate and normalized to the level of GAPDH (glyceraldehyde-3-phosphate dehydrogenase) mRNA. The fluorescent signals were collected during the extension phase, *C*t (threshold cycle) values of the sample were calculated, and transcript levels were analysed by the 2-ΔΔCt method.

**Table 1:** Primers for real-time PCR analysis

Gene name	Chain	Sequence (5′-3′)	Product (bp)
GAPDH	FP RP	AGAAGGCTGGGGCTCATTTG AGGGGCCATCCACAGTCTTC	238
EP2	FP RP	ATACTTAGGCCACCGGTCCT TGAAGCGCATCCTCACAACT	153
FN	FP RP	AATGGAAAAGGGGAATGGAC CTCGGTTGTCCTTCTTGCTC	244
Col I	FP RP	GAGCGGAGAGTACTGGATCG GTTCGGGCTGATGTACCAGT	142
CTGF	FP RP	CAAAGCAGCTGCAAATACCA GGCCAAATGTGTCTTCCAGT	220
COX2	FP RP	AGAAGGAAATGGCTGCAGAA GCTCGGCTTCCAGTATTGAG	194
mPGES-1	FP RP	CGCGGTGGCTGTCATCA AGGGTTGGGTCCCAGGAAT	205

### Western blot analysis

Immunoprecipitation cell lysis buffer was added to the wells, and the plate was put on ice for 30 min, then cells treated as described above were scraped, and the cell lysate was removed into 1.5 ml EP tubes and spun for 15 min. The supernatant was taken for the experiment. Protein concentrations were determined by an optical density (Eppendorf). Proteins were separated by 10% PAGE and then transferred onto PVDF membranes (Millipore) at 350 mA for 2 h, which was later soaked for 2 h on a blocking solution [Tris-buffered saline containing 5% (w/v) non-fat dried milk powder and 0.01% (v/v) Tween-20]. Membranes were incubated for 18 h at 4 °C with rabbit monoclonal primary antibody directed against FN, Col I and CyclinD1(Cayman Chemicals), rabbit monoclonal primary antibody against CTGF and COX-2(Abcam), rabbit anti-ERK, p-ERK, p38MAPK, p-p38MAPK, JNK, p-JNK, CREB1 (cAMP responsive element binding protein 1) and p-CREB1(Cell Signaling Technology, Inc.), diluted 1:1000 in PBS. Anti-β-actin mouse monoclonal antibody (Sigma) was used as an internal control. After incubation, the membrane was washed three times, and peroxidase-conjugated goat anti-rabbit or goat anti-mouse secondary antibodies (ICN Laboratories; diluted 1:10000) were added and incubated for an additional one hour. Reaction was visualized by the ECL (enhanced chemiluminescence) detection system (Pierce) on radiographic films (Kodak) on BIO-RAD ChemiDoc XRS (Bio-Rad). The results were analysed using Image J software.

### Statistical analysis

All experiments were performed in triplicate and the results were expressed as means±S.E. All data were analysed with SPSS19.0 statistical software using Student's *t*-test. Two-tailed *P* values of <0.05 were considered statistically significant.

## RESULTS

### siRNA screen

**Figure 1:**
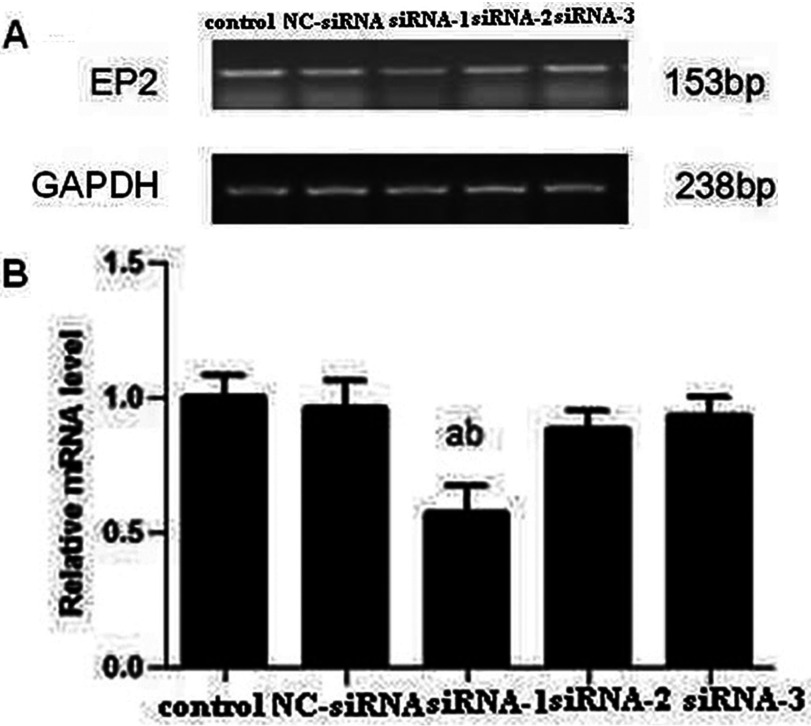
Screening of siRNAs targeting EP2 mRNA. Among the three siRNAs, siRNA-1 showed more intense effect on suppressing EP2 (**A**) RT–PCR; (**B**) Quantification of EP2 expression is achieved using densitometric values normalized to GAPDH levels (a*P*<0.01 versus control group; ^b^*P*<0.05 versus NC-siRNA group).

**Figure 2:**
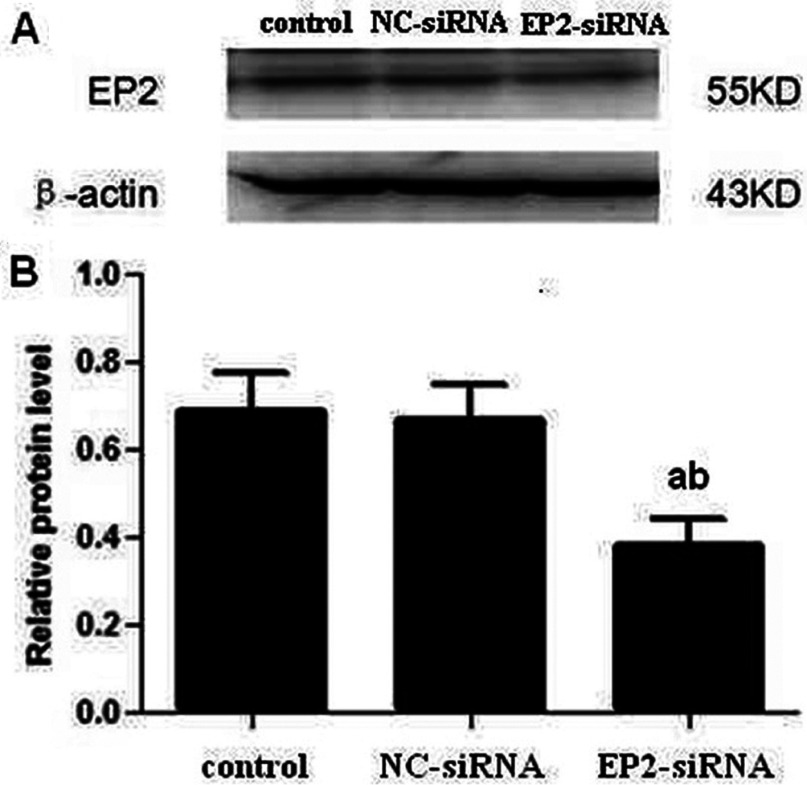
Expression of EP2 in different treated mouse MCs (**A**) Expression of EP2 in EP2-siRNA transfected MCs was detected by Western blot. (**B**) Quantification of EP2 expression is achieved using densitometric values normalized to β-actin levels (a*P*<0.01 versus control group; ^b^*P*<0.05 versus NC-siRNA group).

Three candidate siRNAs, siRNA-1, siRNA-2 and siRNA-3, were transfected into MCs and after 48 h, RT–PCR assay was performed, which showed that a selective 42.3% decrease in EP2 mRNA abundance (Figure 1) compared with control group was confirmed in siRNA-1 transfected MCs. Compared with control group, siRNA-2 and siRNA-3 did not block the expression of EP2 mRNA significantly. The expression of EP2 mRNA in control and NC-siRNA group showed no significant difference, thus excluding the effect of transfection reagent. The results suggest that siRNA-1 is specific and selective against EP2. We selected siRNA-1 for subsequent experiments and named it EP2-siRNA. As shown in Figure 2, Western blot showed a significant reduction in EP2 receptor expression of EP2-siRNA transfected cells as compared with control and NC-siRNA transfected cells.

### Increased expression of EP2 are found in AD-EP2 transfected MCs

**Figure 3:**
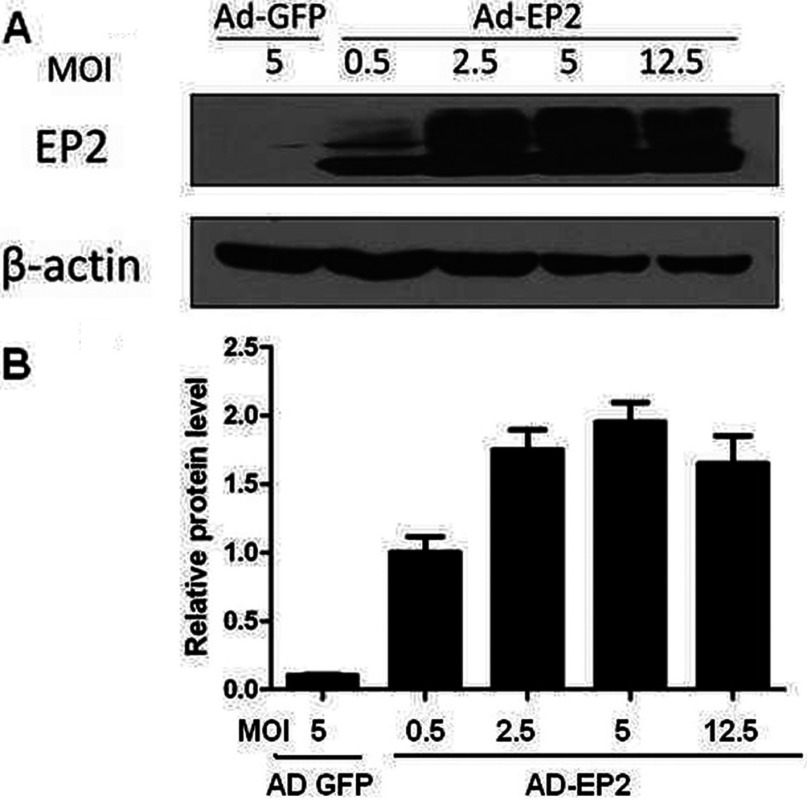
Expression of EP2 in AD-EP2 infected mouse MCs Primary mouse MCs were infected with AD-GFP or different concentration of AD-EP2 (MOI=0.5, 2.5, 5, 12.5) for 24 h and then EP2 protein level was detected by Western blot analysis, β-actin was used as control. (**A**) Expression of EP2 in AD-EP2 infected MCs was detected by Western blot. (**B**) Quantification of EP2 expression is achieved using densitometric values normalized to β-actin levels.

Western blot analysis was done to confirm up-regulation of EP2 expression in AD-EP2 transfected MCs. Our observation showed that AD-EP2 mediated high expression of EP2 and when MOI=5 the AD-EP2 had maximal overexpression effect (Figure 3). Thus 5 MOI is the optimum transfection index and it was selected for subsequent experiments.

### EP2 deficiency resulted in increased MCs proliferation

**Figure 4:**
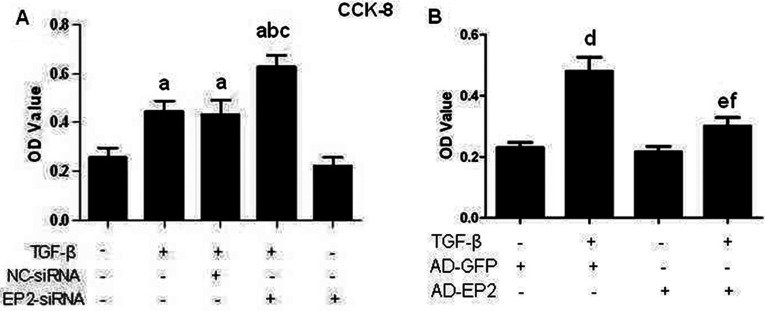
The proliferation of EP2-siRNA or AD-EP2 transfected MCs CCK-8 detected cell proliferation of each experimental group at 24 h after 10 ng/ml TGFβ1 treatment. (**A**) The EP2-siRNA strongly enhanced MCs proliferation, (**B**) Overexpression of EP2 by AD-EP2 suppressed proliferation of MCs (a*P*<0.05 versus control; ^b^*P*<0.05 versus TGFβ1 group; ^c^*P*<0.05 versus NC-siRNA+TGFβ1group; ^d^*P*<0.05 versus AD-GFP group;e *P*<0.05 versus AD-EP2 group; ^f^ *P*<0.05 versus AD-GFP+TGFβ1group).

To determine whether TGFβ1 was able to affect the proliferation of MCs, a CCK-8 assay was performed on cells after they were treated with 10 ng/ml TGFβ1 for 24 h (Figure 4). The results showed that the proliferative capacity of MCs was significantly increased by 10 ng/ml TGFβ1 (*P*<0.05). Further, we investigated the role of EP2 in the proliferation of MCs. The EP2-silencing RNA strongly enhanced MCs proliferation (Figure 4A), while overexpression of EP2 by AD-EP2 suppressed the proliferation of MCs (Figure 4B), demonstrating that EP2 activation mediates MCs proliferation.

### EP2 deficiency reduced PGE_2_ and cAMP levels in MCs

**Figure 5:**
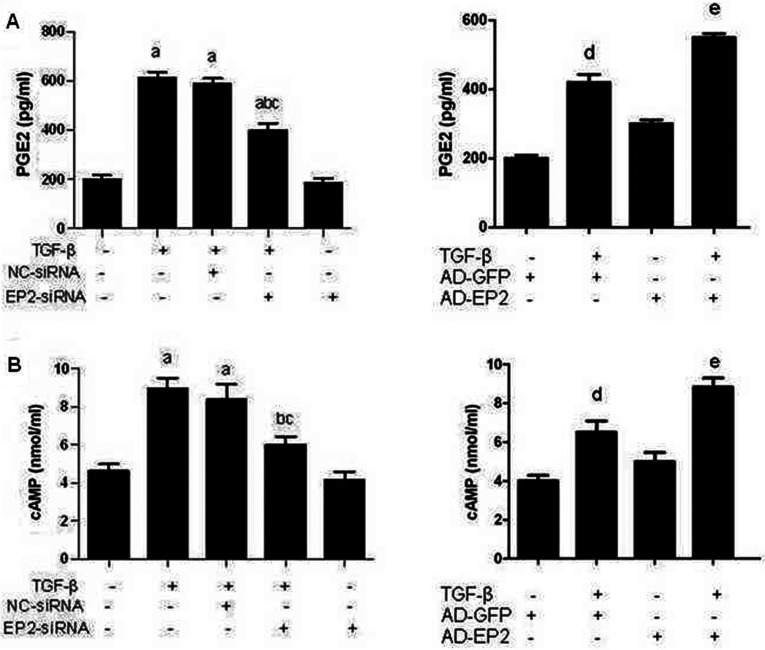
The productivity of PGE_2_ and cAMP of MCs of each experimental group ELISA detected the productivity of PGE_2_ and cAMP of each experimental group at 24 h after 10 ng/ml TGFβ1 treatment. (**A**) PGE_2_ levels in EP2-siRNA+ TGFβ1 group were greatly reduced compared with NC-siRNA+TGFβ1 group, while they were significantly increased in AD-EP2+TGFβ1 group compared with AD-GFP+TGFβ1 group. (**B**) TGFβ1-induced cAMP production was consistent with PGE_2_ (a*P*<0.05 versus control; ^b^*P*<0.05 versus TGFβ1group; ^c^*P*<0.05 versus NC-siRNA+TGFβ1group; ^d^*P*<0.05 versus AD-GFP group; e*P*<0.05 versus AD-GFP+TGFβ1group).

EP2 receptor-dependent signalling leads to an increase in cAMP levels [13]. cAMP is an important second messenger in the EP2-mediated signalling pathway. Thus, we hypothesized that cAMP levels would be reduced in the supernatant of EP2-siRNA transfected MCs compared with control MCs following 10 ng/ml TGFβ1 treatment. As expected, TGFβ1 treatment resulted in a high induction of PGE_2_ and cAMP in control MCs. However, PGE_2_ and cAMP levels in EP2-siRNA transfected MCs were greatly reduced, while they were increased in AD-EP2 transfected MCs (Figure 5). This suggests that TGFβ1 stimulates the production of PGE_2_ and this activates EP2 receptor, which can induce cAMP production and that cAMP-dependent PKA (protein kinase A) signalling may affect expression of genes related to cell proliferation, apoptosis and fibrosis in glomerular.

### EP2 deficiency increased the expression of FN, Col 1, CTGF and CyclinD1

**Figure 6:**
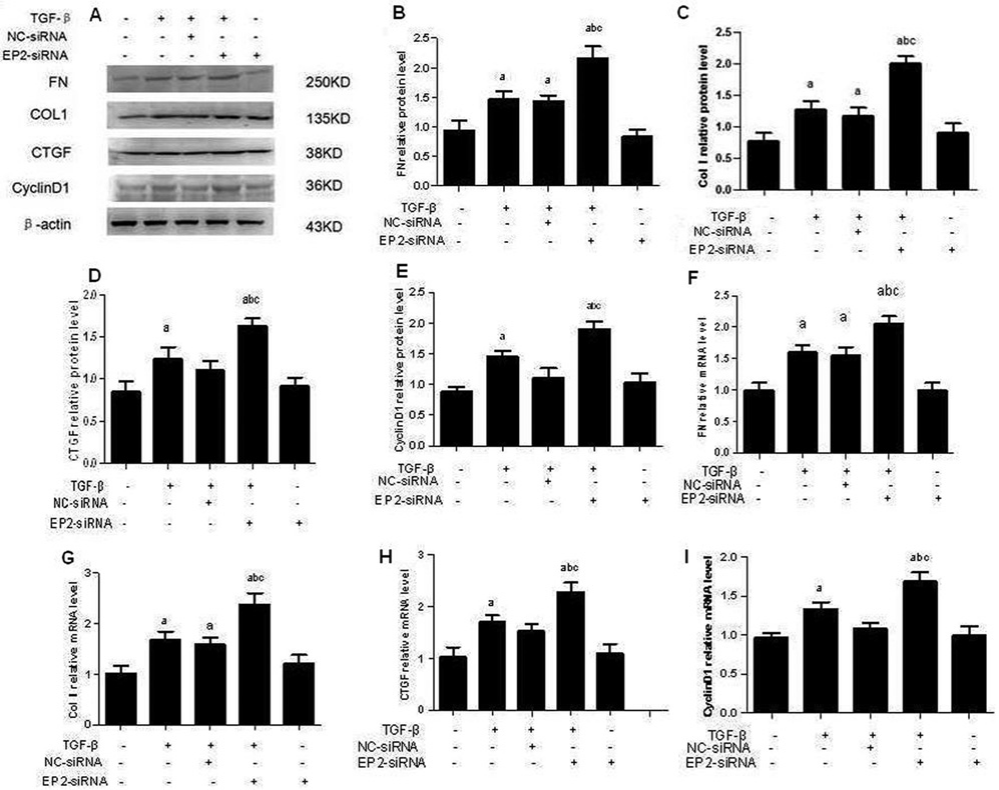
The expression of FN, Col I, CTGF and CyclinD1 in EP2-siRNA transfected MCs Western blot detected the expression of FN, Col I, CTGF and CyclinD1 of each experimental group at 24 h after 10 ng/ml TGFβ1 treatment. The expressions of those proteins of EP2-siRNA+TGFβ1 group were increased significantly than that of NC-siRNA+TGFβl group. (**A**) Western Blot. (**B**–**E**) Quantification of FN, Col I, CTGF and CyclinD1 expression is achieved using densitometric values normalized to β-actin levels. (**F**–**I**) The relative mRNA levels of FN, Col I, CTGF and CyclinD1 are also shown (a*P*<0.05 versus control; ^b^*P*<0.05 versus TGFβ1 group; ^c^*P*<0.05 versus NC-siRNA+TGFβ1group).

**Figure 7:**
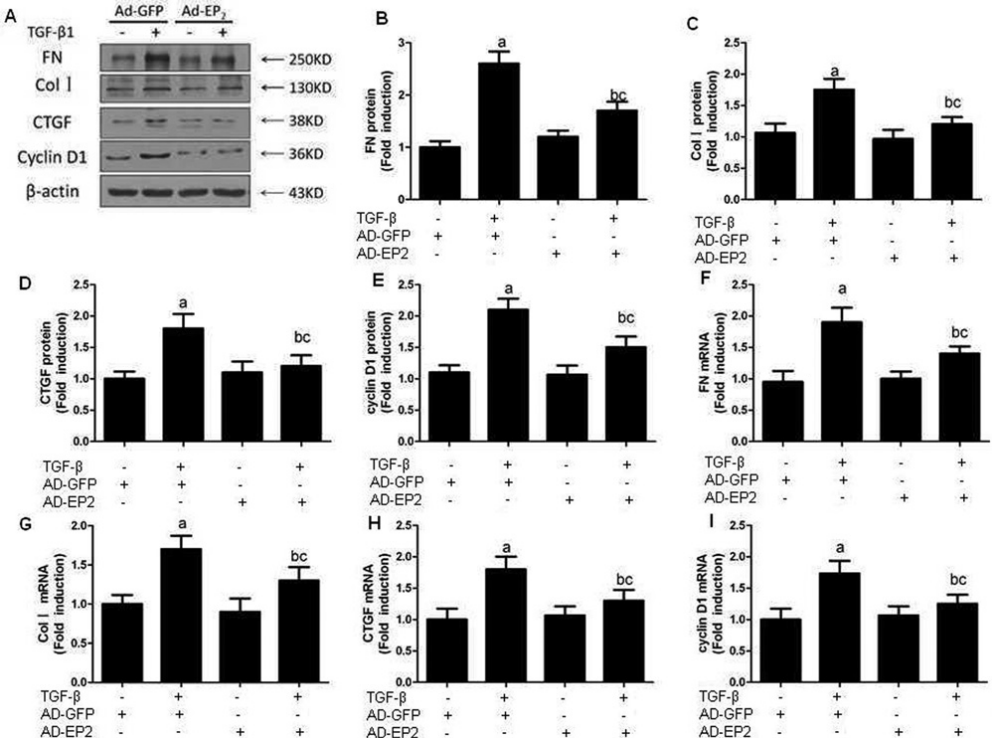
The expression of FN, Col I, CTGF and CyclinD1 in AD-EP2 infected MCs Western blot detected the expression of FN, Col I, CTGF and CyclinD1 of each experimental group at 24 h after 10 ng/ml TGFβ1 treatment. The expressions of those proteins of AD-EP2+TGFβ1 group were decreased significantly than that of AD-GFP+TGFβ1 group. (**A**) Western blot. (**B**–**E**) Quantification of FN, Col I, CTGF and CyclinD1 expression is achieved using densitometric values normalized to β-actin levels. (**F**–**I**): The relative mRNA levels of FN, Col I, CTGF and CyclinD1 are also shown (a*P*<0.05 versus AD-GFP group; ^b^*P*<0.05 versus AD-EP2 group; ^c^*P*<0.05 versus AD-GFP+TGFβ1group).

The accumulation of glomerular ECM is one of the critical pathological characteristics of renal fibrosis. FN and Col I are important constituents of ECM. The expressions of FN, Col I, CTGF and CyclinD1 were assessed by Western blot and RT–PCR. After the MCs were stimulated by 10 ng/ml TGFβ1 for 24 h, the expressions of FN, Col I, CTGF and CyclinD1 protein of MCs were both increased than the untreated MCs (*P*<0.05). Furthermore, the expressions of FN, Col I, CTGF and CyclinD1 protein of EP2-siRNA transfected MCs were increased significantly than that of the control MCs (*P*<0.05) (Figure 6). MCs infected with AD-EP4 inducing a significant decrease in FN, Col I, CTGF and CyclinD1 expression than AD-GFP infected controls (*P*<0.05; Figure 7). The results obtained by RT–PCR were consistent with that obtained by Western blot. The results showed that the expression of FN, Col I, CTGF and CyclinD1 changed with the expression of EP2, they were decreased with overexpression of EP2 and increased with down-regulation of EP2. The data suggest that the EP2 receptor may play an important role in TGFβ1-induces ECM accumulation.

### EP2 deficiency increased the expression of enzymes involved in the TGFβ1-induced synthesis of PGE_2_

**Figure 8:**
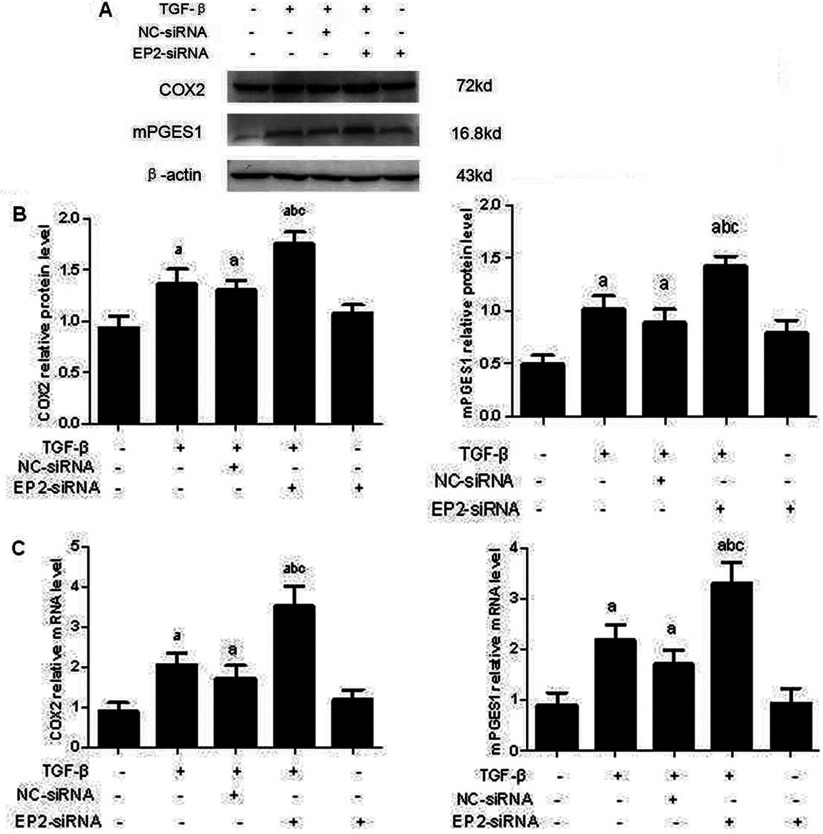
The expression of COX2 and mPGES1 in EP2-siRNA transfected MCs Primary MCs were transfected with EP2-siRNA for 12 h and then incubated with 10 ng/ml TGFβ1 for 24 h. The expression of COX2 and mPGES1 in EP2-siRNA+TGFβ1 group were significantly increased than that of NC-siRNA+TGFβ1group. (**A**) Western blot (**B**) Quantification of COX2 and mPGES1 expression is achieved using densitometric values normalized to β-actin levels. (**C**) The relative mRNA levels of COX2 and mPGES1 are also shown (a*P*<0.05 versus control; ^b^*P*<0.05 versus TGFβ1 group; ^c^*P*<0.05 versus NC-siRNA+TGFβ1group).

**Figure 9:**
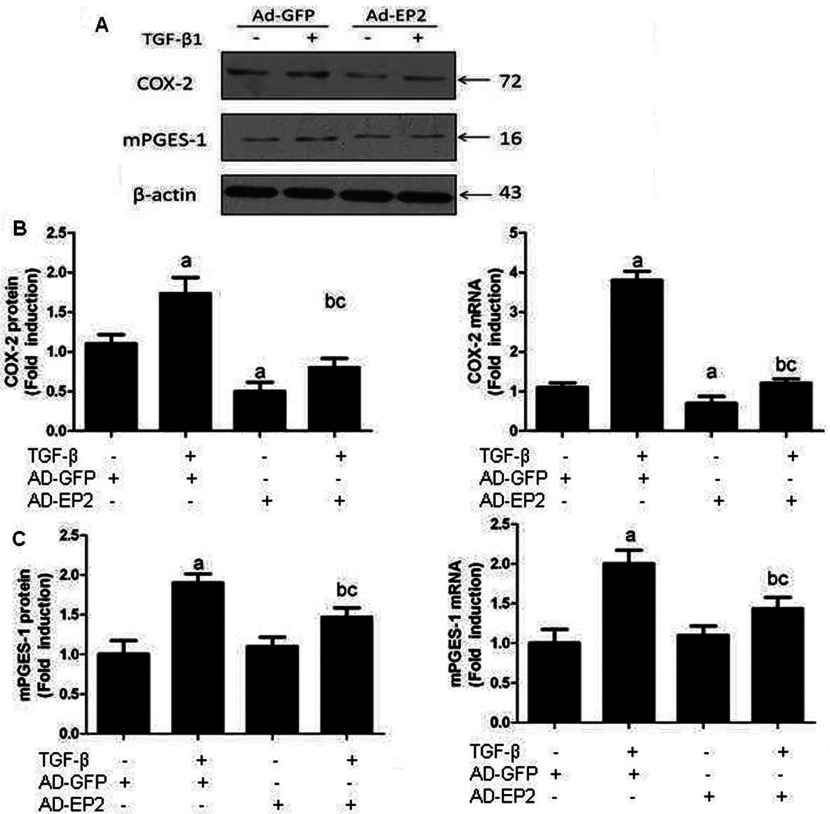
The expression of COX2 and mPGES1 in AD-EP2 infected MCs Primary MCs were transfected with AD-EP2 for 24 h and then incubated with 10 ng/ml TGFβ1 for 24 h. The expression of COX2 and mPGES1 in AD-EP2+TGFβ1group were significantly decreased than that of AD-GFP+TGFβ1group. (**A**) Western blot. (**B**) Quantification of COX2 and mPGES1 expression is achieved using densitometric values normalized to β-actin levels. (**C**) The relative mRNA levels of COX2 and mPGES1 are also shown (a*P*<0.05 versus AD-GFP group; ^b^*P*<0.05 versus AD-EP2 group; ^c^*P*<0.05 versus AD-GFP+TGFβ1group).

PGE_2_ has been shown to amplify its own production by inducing COX2 expression in various cells [14]. It has been demonstrated that TGFβ induces COX2 expression and subsequent PGE_2_ production and that up-regulated COX2 inhibits Smad3 activation during breast cancer progression [15]. Unfortunately, the components and effectors lying downstream of COX2-PGE_2_ that mediated their anti-TGFβ activities remain unknown. We hypothesized that EP2 receptor may regulate COX2 expression through a positive feedback. Consistent with previous study, TGFβ stimulation induced significant COX2 and mPGES1 expression in MCs. To determine the effect of EP2 repression on COX2 and mPGES1 induction, MCs were topically transfected with EP2-siRNA or AD-EP2 to decrease or increase EP2 expression. COX2 and mPGES1 expression was assessed at both the mRNA and the protein levels, as shown in Figures 8 and 9. EP2 deficiency increased COX2 and mPGES1 expression, but a significant decrease was observed in AD-EP2 transfected MCs compared with AD-GFP-transfected MCs. These data suggest that COX2 and mPGES1 expression can be regulated by the EP2-signalling pathway.

### EP2 deficiency increased p38MAPK and ERK1/2 phosphorylation

**Figure 10:**
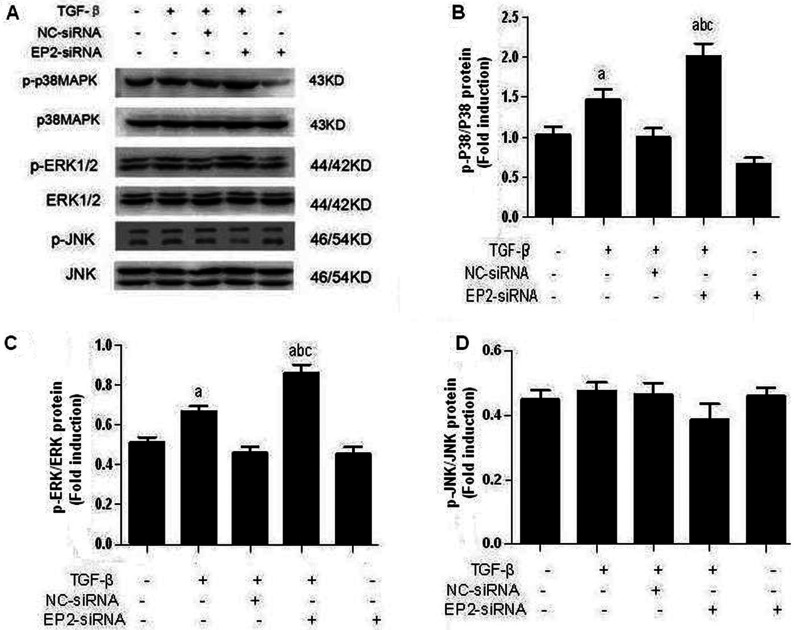
The expression of p-p38MAPK, p-ERk and p-JNK in EP2-siRNA-transfected MCs. Primary MCs were transfected with EP2-siRNA for 12 h and then incubated with 10 ng/ml TGFβ1 for 24 h The expression of p-p38MAPK, p-ERK and p-JNK in EP2-siRNA+TGFβ1group were significantly increased than that of NC-siRNA+TGFβ1group. (**A**) Western blot. (**B**–**D**) Quantification of p-p38MAPK, p-ERk and p-JNK expression is achieved using densitometric values normalized to β-actin levels (a*P*<0.05 versus control; ^b^*P*<0.05 versus TGFβ1group; ^c^*P*<0.05 versus NC-siRNA+TGFβ1group).

**Figure 11:**
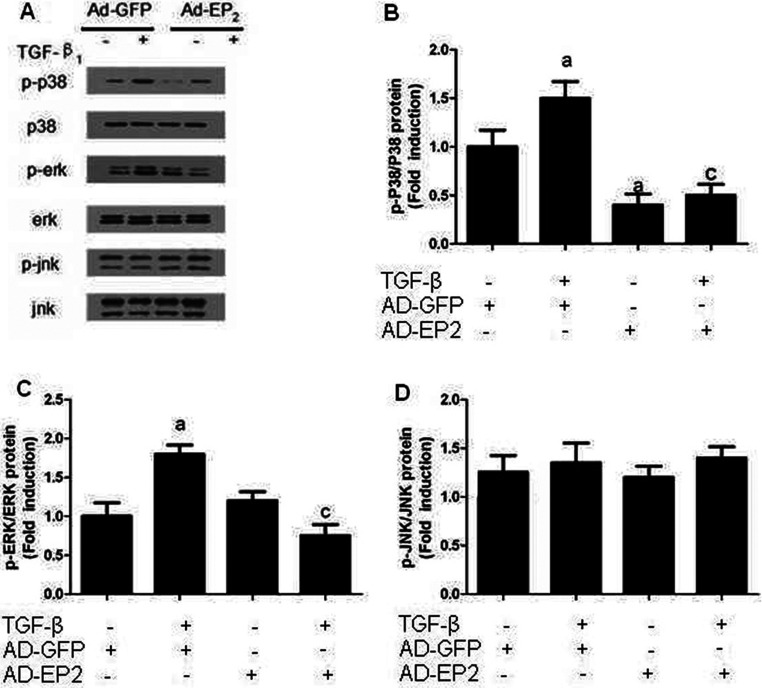
The expression of p-p38MAPK, p-ERk and p-JNK in AP-EP2-infected MCs Primary MCs were infected with AP-EP2 for 24 h and then incubated with 10 ng/ml TGFβ1 for 24 h. The expression of p-p38MAPK, p-ERK and p-JNK in AD-EP2+TGFβ1 group were significantly decreased than that of AD-GFP+TGFβ1 group. (**A**) Western Blot. (**B**–**D**) Quantification of p-p38MAPK, p-ERK and p-JNK expression is achieved using densitometric values normalized to β-actin levels (a*P*<0.05 versus AD-GFP group; ^c^*P*<0.05 versus AD-GFP+TGFβ1 group).

Because phosphorylation of Akt [also known as PKB (protein kinase B)] and ERK1/2 has been suggested to mediate the growth-promoting effect of PGE_2_ in many cancer cell types [16,17], we examined the direct effects with TGFβ on the phosphorylation of these proteins. As shown in Figures 10 and 11, treatment of TGFβ significantly stimulated the phosphorylation of p38MAPK and ERK1/2. To further examine whether the EP2 receptor is involved in mediating the p38MAPK and ERK1/2 phosphorylation. Western blot analysis has shown that siRNA-EP2 markedly increased p38MAPK and ERK1/2 phosphorylation while AD-EP2 significantly decreased p38MAPK and ERK1/2 phosphorylation after 24 h treatment. Moreover, Western blot analysis revealed that the phosphorylation of Jun, member of the MAPK family to which ERK1/2 belongs, was not affected by neither TGFβ nor EP2 receptor. The data indicated that EP2 may inhibit TGFβ1-induced MCs proliferation and ECM accumulation via down-regulation of p38MAPK and ERK1/2 phosphorylation.

### EP2 deficiency increased CREB1 phosphorylation

**Figure 12:**
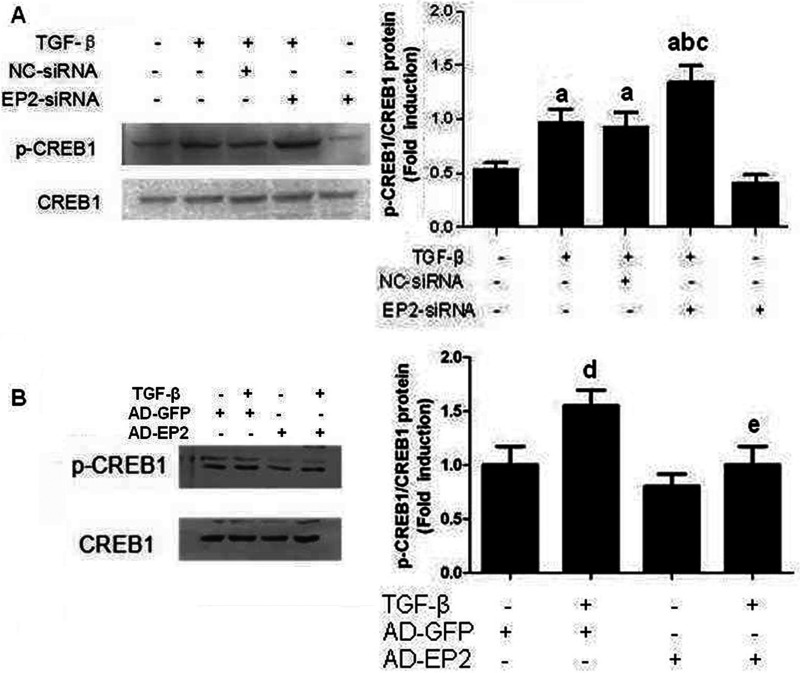
Effect of treatment with siRNA or adenoviruses targeting EP2 on expression of p-CREB1 (**A**) Primary MCs were transfected with EP2-siRNA for 12 h and then incubated with 10 ng/ml TGFβ1 for 24 h. The expression of p-CREB1 in EP2-siRNA+TGFβ1 group were significantly increased than that of NC-siRNA+TGFβ1 group. (**B**) Primary MCs were infected with AP-EP2 for 24 h and then incubated with 10 ng/ml TGFβ1 for 24 h. The expression of p-CREB1 in AD-EP2+TGFβ1 group were significantly decreased than that of AD-GFP+TGFβ1 group (a*P*<0.05 versus control; ^b^*P*<0.05 versus TGFβ1 group; ^c^*P*<0.05 versus NC-siRNA+TGFβ1group; ^d^*P*<0.05 versus AD-GFP group; e*P*<0.05 versus AD-GFP+TGFβ1 group).

We previously found that EP2 deficiency had significantly reduced cAMP production in the MCs supernatant following treatment with TGFβ1 *in vivo* compared with that in control MCs supernatant. This led us to hypothesize that activation of the EP2/cAMP/PKA-signalling pathway would led to phosphorylation of CREB. We found that EP2—siRNA-transfected MCs have increased CREB phosphorylation compared with those of NC—siRNA-transfected MCs following TGFβ1 treatment (Figure 12A). EP2 overexpression in MCs significantly reduced TGFβ1-induce CREB phosphorylation (Figure 12B). Collectively, this study suggests that EP2 regulates CREB phosphorylation via cAMP/PKA signalling.

## DISCUSSION

In several studies, a role for PGs in renal fibrosis has been shown [18,19]. In the nephrotoxic mercury chloride [HgCl(2)] rat model of acute kidney failure, although the EP2 or the EP2/4 agonist did not change the serum creatinine values, the EP2 receptor agonist increased the survival rate. These findings suggest that PGE_2_ has an important role in acute kidney failure via the EP2 receptor [20]. Other studies demonstrate that PGE_2_ inhibits transition of fibroblasts to myofibroblasts by an EP2 receptor-activated pathway [21]. There is limited and conflicting data on the role and distribution of EP2 receptor on MCs [22,23].

Based on previous observations, we hypothesized that activation of the EP2 receptor inhibits the progression of MCs damage induced by TGFβ1. To investigate this hypothesis, we screened EP2 siRNA which best reduced the expression of EP2 in MCs and delineated the role of EP2 on TGFβ1-induced MCs damage. We found that EP2 silencing in MCs increased the proliferation of MCs and the expression of FN, Col I,mRNA and protein induced by TGFβ1. This result indicates that the EP2 receptor may be involved in the progression of MCs proliferation and may inhibit TGFβ1-induced renal fibrosis. To evaluate this, we also overexpressed EP2 receptor by adenovirus technology. Up-regulation of EP2 in MCs significantly inhibited TGFβ1-induced MCs proliferation and decreased the expression of FN, Col I, mRNA and protein. These results indicate that increased expression of EP2 may have an inhibitory action on TGFβ1-induced renal fibrosis and inhibit the proliferation of MCs. Similar results were obtained by Kolodsick [21], who found that PGE_2_ inhibits TGFβ1-induced pulmonary fibrosis by an EP2 receptor-activated pathway.

It is accepted that both TGFβ1 and one of its downstream effectors CTGF (connective tissue growth factor) play roles in regulating the pathogenesis of this fibrotic kidney disease [24]. CTGF is an established effector of TGFβ1 driven responses in diabetic nephropathy. Cyclin D1 is considered as an oncogene and can promote progression of the cell cycle from G1 to S phase by CDK4/CDK6 (cyclin D-dependent kinases)-mediated phosphorylation of the Rb (retinoblastoma) protein [25–27]. Our study showed that silencing of EP2 receptor increased CTGF and Cyclin D1 production induced by TGFβ1, whereas the up-regulation of EP2 by adenovirus decreased it, demonstrating that activation of EP2 decreases the expression of CTGF and Cyclin D1, prevent S phase entry and inhibit the proliferation of MCs.

It has been reported that COX-2 is constitutively expressed in the mammalian kidney and that its metabolites, including prostanoids, play an integral role in the regulation of renal haemodynamics, the RAS (renin–angiotensin system), nephronogenesis and renal pathogenesis [28]. mPGES-1 (membrane-associated prostaglandin E1) is inducible by similar stimuli that induce COX-2 and appears to be functionally coupled with COX-2 and its induction is usually co-ordinated with COX-2 [29]. Our results have shown that EP2 deficiency in MCs augmented COX2 and mPGES-1 expression stimulated by TGFβ1, whereas EP2 over expression decreased their expression. The COX2/mPGES-1/PGE_2_ pathway is critical in MCs damage progression. Furthermore, overexpression of mPGES-1 was able to induce the activity of EP2 and PGE_2_. We detected the expression of PGE_2_ in TGFβ1 treated MCs. Our ELISA assay showed that TGFβ1 treatment led to augmented PGE_2_ production. Moreover, elevated EP2 expression up-regulates the production of PGE_2_ while lack of EP2 expression decreased PGE_2_ production. Taken together these data suggest that the treatment of TGFβ1 increased the expression of COX-2 and mPGES-1, resulting in an augmented PGE production and activation of EP2 receptor, which then decreases the expression of both COX-2 and mPGES-1. This feedback regulation of the PGE_2_-synthesizing enzymes COX-2 and mPGES-1 in MCs inhibited the proliferation of MCs and accumulation of ECM, mitigated MCs damage and prevented renal fibrosis. One important contribution of this study is that PGE_2_ plays a useful role in preventing MCs damage via EP2 receptor.

It has been reported that PGE_2_ inhibits TGFβ1-induced transition of fibroblasts to myofibroblasts by increasing cAMP levels via EP2 receptor [21]. In the present study, we have shown that treatment of MCs with TGFβ1 induced FN and Col I expression, resulting in augmented cAMP production. EP2 deficiency in MCs down-regulates the expression of cAMP induced by TGFβ1, suggesting an enhancement of the fibrosis effect. In contrast, overexpression of EP2 significantly increased the production of cAMP, suggesting that although activation of EP2 receptor cannot entirely inhibit TGFβ1 induced renal fibrosis, it can decrease cell damage via up-regulation of the cAMP levels.

The inhibition of cell proliferation appears to involve changes in MAPK signalling, particularly in the ERK 1/2 pathway [30]. This signalling pathway plays an important role in the growth, development, proliferation and malignant transformation of cells, with ERK 1/2 as the key molecules [31]. The CREB is an activity regulated transcription factor that modulates the transcription of genes with CRE (cAMP responsive element) located in their promoter regions. Previous studies have found that the activation of ERK is crucial for cell cycle progression and migration. Furthermore, stimulation of cAMP/PKA pathway inhibited S phase entry in MCs inhibit phosphorylation of CREB [32].

The present study shows that TGFβ1 treatment of MCs induced phosphorylation of ERK and p38 and lead to downstream activation of CREB1. Deficiency of EP2 expression led to increases in the levels of p-ERK 1/2, p-p38 and p-CREB1 and that their activation was decreased by high levels of expression of EP2. However, EP2 expression did not affect Jun phosphorylation, which may relate to the cell type. The results suggested that EP2 stimulation causes inhibition of TGFβ1-induced phosphorylation of ERK, p38 and CREB1. Furthermore, the inhibition of MAPKs/CREB pathway prevents the TGFβ1-induced accumulation of ECM.

In summary, the results of the present study indicate that the stimulation of EP2 receptor inhibits MAPK/CREB pathway and its downstream effectors, cell cycle proteins and CTGF via an increase in the level of cAMP, thus contributing to inhibition of TGFβ1-induced MCs proliferation and accumulation of ECM.
